# THE PUBLIC HEALTH IMPLICATIONS OF DEMENTIA CAREGIVING IN THE US: FINDINGS FROM THE 2021 BRFSS

**DOI:** 10.1093/geroni/igad104.0817

**Published:** 2023-12-21

**Authors:** Joseph Gaugler, Raza Lamb, Ben Denno, Matthew Baumgart, Hawking Yam, Lisa McGuire

**Affiliations:** University of Minnesota, Minneapolis, Minnesota, United States; Alzheimer’s Association, Chicago, Illinois, United States; Alzheimer’s Association, New York, New York, United States; Alzheimer’s Association, New York, New York, United States; University of Minnesota, Minneapolis, Minnesota, United States; CDC, Atlanta, Georgia, United States

## Abstract

To further elevate dementia caregiving as a public health imperative, representative data that highlights more local variations in dementia caregiving is essential, as such data can better target and tailor services as well as advance policy innovations at the state level. We empirically examined state variations in dementia caregiving in the U.S. by analyzing data from the Behavioral Risk Factor Surveillance System (BRFSS) among the 39 states that used the Caregiver Module in 2021. Prevalence of hypertension among dementia caregivers ranged from a low of 28.7% (CI 16.7-44.8) in South Dakota to a high of 53.6% (CI 43.9-63.1) in Arkansas. The mean hypertension prevalence rate in the 39 states for dementia caregivers was 42.0%, compared with a mean hypertension prevalence rate for non-dementia caregivers of 38.2%. Close to half (44.2%, CI 31.8-56.6) of dementia caregivers in Michigan provide care for 40 or more hours per week. In 35 of the 39 states, at least one-third of dementia caregivers provide care for 20 or more hours per week; in comparison, at least one-third of non-dementia caregivers provide care for 20 or more hours per week in only 7 of the 39 states. The findings illustrate how intensity and the health implications of dementia care vary across states in the U.S. and can help inform more localized programmatic and policy targets to address the public health implications of dementia care.

